# Hand Digit Revascularization: Could Be an “Elective-Urgence” Surgery?

**DOI:** 10.3390/jcm13175120

**Published:** 2024-08-29

**Authors:** Francesco De Francesco, Olimpia Mani, Pasquale Gravina, Michele Riccio

**Affiliations:** Department of Reconstructive Surgery and Hand Surgery, Azienda Ospedaliera Universitaria delle Marche, 60126 Ancona, Italy; olimpia.mani@ospedaliriuniti.marche.it (O.M.); pasquale.gravina@ospedaliriuniti.marche.it (P.G.); michele.riccio@ospedaliriuniti.marche.it (M.R.)

**Keywords:** revascularization, delayed replantation, amputation, ischemia time, functional outcomes, hand trauma

## Abstract

**Background**: A continuous obstacle that has limited access to and implementation of finger replantation surgery is timeliness, as ischemia time is traditionally considered a crucial factor for success. However, claims that the vitality of amputated fingers decreases after 6 h of warm ischemia and 12 h of cold ischemia are mostly based on theoretical considerations. **Methods**: Here we present a case of multi-digit revascularization after 72 h of warm ischemia using the microsurgical arteriovenous bypass technique. **Results**: In the reported case, revascularization was performed after a long ischemic period and showed good recovery of motor and sensory function. **Conclusions**: We identified significant limitations in the literature supporting time limits of ischemia and recent evidence demonstrating the feasibility of delayed finger replantation. The current treatment approach for amputation injuries often requires transfers or nighttime emergency procedures, increasing costs and limiting the national availability of finger replantation. Changes to finger replantation protocols based on evidence could expand access to this service and improve the quality of care.

## 1. Introduction

There is no common consensus in the medical literature or among professionals regarding the maximum ischemia time in digit replantation or hypoperfusion time in revascularization. The issue of ischemia time is critical as it plays a significant role in determining the success of the surgical intervention and the survival of the replanted/revascularized tissue. In 1975, Hayhurst and colleagues conducted experiments demonstrating that amputated segments could tolerate a warm ischemia time limit of 6 h and a cold ischemia time limit of 24 h for successful digital replantation [1]. These values have become a kind of reference standard for many years. However, they are based on experimental conditions and may not take into account all the clinical variables present in real cases. Ever-increasing case-reports of delayed digital replantation and revascularization weakened this dogma [2,3,4,5,6,7,8]. Increasing case reports of delayed replantation have challenged the rigid limits imposed by initial studies, highlighting that with the right medical and technical interventions, positive outcomes are possible even in seemingly unfavorable conditions. Advancements in surgical techniques, progress in microsurgery, and optimization of medical therapies have significantly increased the chances of success. New tissue preservation techniques, the use of vasodilators and antithrombotic drugs, and better postoperative care have extended the “window” of time for successful intervention. Standardizing a maximum time for replantations or revascularizations is complex due to various factors and variables influencing both the success and failure of the procedures. These variables include the nature and extent of the trauma, the patient’s physiological conditions, the presence of comorbidities, the time and conditions of transport of the amputated segment, timely intervention, and the quality of medical support immediately after the trauma. Some specific factors that further complicate standardization include: 1. ** Type of Injury:** Clean cuts tend to recover better compared to crush or avulsion injuries, which can cause more extensive damage to soft tissues and nerves. 2. ** Patient’s Condition:** Patients with pre-existing conditions such as diabetes or atherosclerosis may have a reduced capacity for healing and restoration of blood flow. 3. ** Segment Preservation:** The way the amputated segment is preserved (e.g., appropriately refrigerated but not frozen) can make a significant difference in the tissue’s viability. 4. ** Timing of Surgery:** Timely surgical intervention and an experienced surgical team are essential for the procedure’s success.

Finally, it is essential to underline the intrinsic difference between replantation and revascularization: while in the first case, it is appropriate to talk about ischemia time, in the second instance, the term hypoperfusion time is more correct. In cases of subamputations, the remaining tissue bridges allow some circulation through communicating vessels and collateral circle variably developed. However, the blood supply is often insufficient, leading to a state of hypoperfusion that over time causes the discoloration and necrosis of the finger.

In light of these variables, it is clear that a “one size fits all” approach is not feasible for every case. Developing a standard maximum time could be theoretically interesting; however, it proves practically challenging without considering the specific circumstances of each individual case. Finally, understanding the hypoperfusion time limits that guarantee a high success rate in digit replantation and revascularization is essential not only to improve clinical outcomes but also for more effective management of emergency resources, which are increasingly scarce in modern healthcare systems.

Our proposal of a case report on delayed digit revascularization, referred to our center 72 h after trauma with warm ischemia of all long fingers of the dominant hand, represents an opportunity to explore and better understand these limits. The reported warm ischemia period is the longest described in the literature, and this experience provided the foundation for conducting a literature review on similar cases and analyzing the potential for performing revascularization of incomplete digit amputation as “elective surgery”.

## 2. Detailed Case Description

### 2.1. Patient History and Initial Presentation

A 51-year-old patient was referred to us from a peripheral orthopedic department with a diagnosis of friction trauma to the left hand. The injury occurred while tightening a rope, resulting in a transverse lacerated-contused wound at the level of the palm, extending from the second to the fifth metacarpophalangeal joint. The incident occurred 72 h prior to the consultation. The patient was referred to us due to observed vascular compromise in the fifth finger and discoloration of the other fingers.

### 2.2. Clinical Examination

During the clinical examination, we found a pattern of decreased blood flow affecting the third, fourth, and fifth fingers of the hand, suggesting significant vascular compromise in these areas. In particular, no spontaneous bleeding was observed when the third, fourth, and fifth fingers were pricked with a needle, an alarming sign that blood flow is severely reduced or completely absent. This lack of spontaneous bleeding is indicative of circulatory insufficiency, due to interruption of the common digital arteries caused by the trauma suffered.

In addition to the compromised blood flow, we observed early signs of skin necrosis in the proximal phalanges of the third and fourth fingers. This suggests that cell death has just begun in these areas, probably due to a prolonged lack of oxygen and nutrients from reduced blood supply. In the fifth finger, the situation is even more severe, with advanced necrosis, indicating that the tissue has been without blood supply for a longer period of time, causing extensive and irreversible tissue damage. Furthermore, there was a total loss of sensation in all long fingers (third, fourth, and fifth fingers), suggesting damage to the peripheral nerves associated with their interruption.

**Figure 1:**
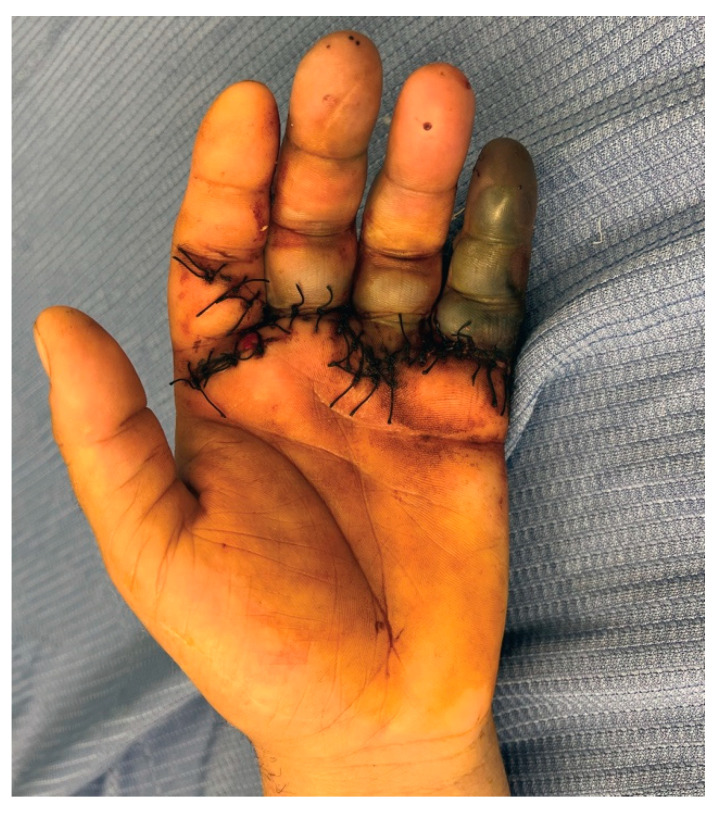
Patient hand the day of centralization in our department. It is evident V ray necrosis and vascular impairment of III and IV finger, with no blood flow at needle prick.

Figure 1 clearly shows the affected areas and the extent of necrosis, providing visual support to our clinical observations and helping outline the most appropriate therapeutic strategy to address these serious vascular and neurological complications.

### 2.3. Surgery

**Figure 2:**
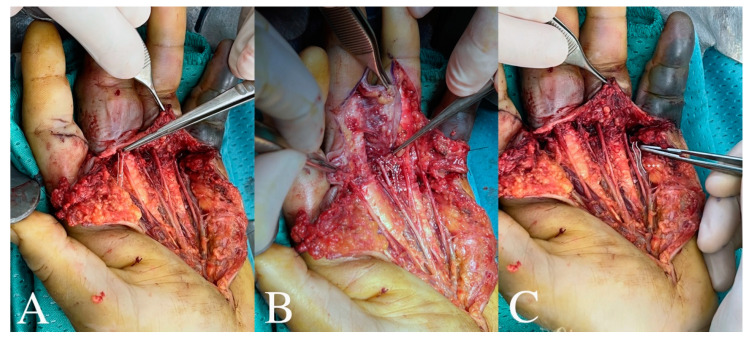
During surgery, it was discovered that there was interruption of (**A**) digital common artery for the second and third fingers (straight line), of (**B**) common digital artery for the third and fourth fingers (dotted line), and (**C**) common digital artery for the fourth and fifth fingers (curved line).

The patient was urgently taken to the operating room for wound exploration. Intraoperatively, we identified an injury to the ulnar collateral digital artery of the second finger, the common digital artery of the third and fourth fingers, and the common digital artery of the fourth and fifth fingers. However, the digital nerves were anatomically continuous (Figure 2).

**Figure 3:**
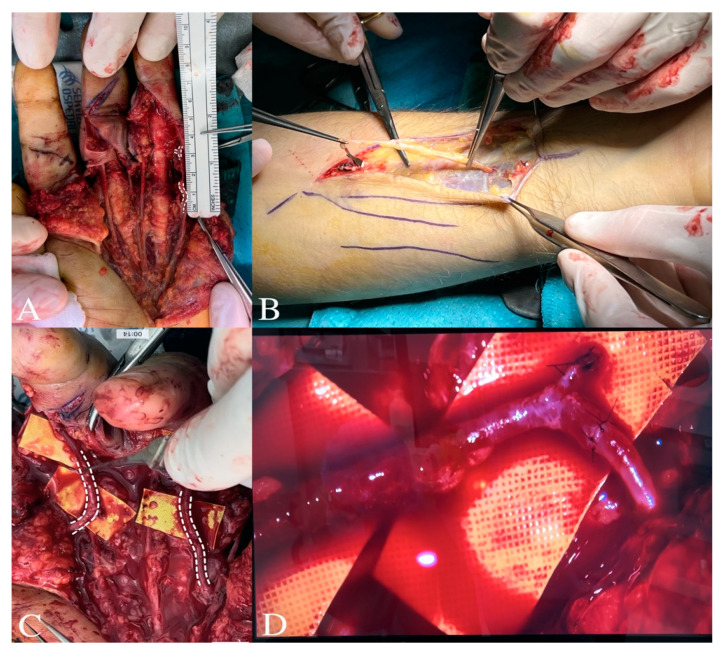
The surgical procedure involved the resection of the arterial stumps until the presence of good intimal tissue was found. This procedure inevitably created a gap in all digital arteries to be reconstructed of about 2–3 cm (**A**). In (**B**), a vein is shown being harvested from the forearm. In (**C**), microsurgical reconstruction of the common digital arteries for the second and third finger, and for the third and fourth finger is shown using an interposed venous bypass. In (**D**), a detailed arterio-venous anastomosis is shown.

We proceeded with the resection of the common digital arteries, leaving a gap of approximately 3 cm. We harvested an appropriately sized vein from the forearm and performed revascularization of the common digital arteries using a venous bypass (Figure 3).

**Figure 4:**
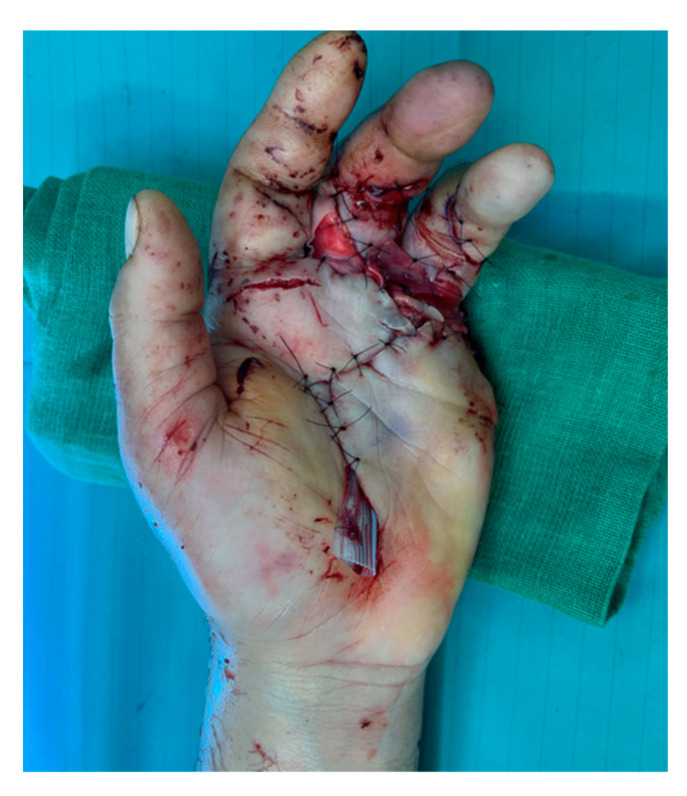
II-III-IV finger vascularized after surgery.

For the necrotic fifth finger, we performed regularization at the metacarpal head. Necrotic areas in the proximal phalanges of the third and fourth fingers were debrided, and an Integra dermal substitute was applied. At the end of the procedure, the revascularized fingers appeared pink, trophic, and vital (Figure 4).

### 2.4. Follow-Up and Outcome

We clinically followed the patient for one year. During follow-up, we assessed hand function using the Brief Michigan Hand Outcomes Questionnaire (MHQ) global score (Table 1). The Brief Michigan Hand Outcomes Questionnaire (MHQ) is a well-regarded tool for assessing hand function [9,10], as it covers various important aspects including pain, function, aesthetics, and patient satisfaction. Monitoring the global score over the course of a year provides valuable insights into the patient’s recovery and overall hand function improvements. The scores of our patient have consistently improved during the follow-up period. Initially, in the first month after the surgical intervention, the overall score was 49, after 6 months from the surgery the score dropped to 31, and finally, after one year, the score further decreased to 18. These data indicate a steady improvement in the patient’s hand function over the course of the year.

At the beginning of the follow-up, the score suggests that the patient may have experienced significant impairment in hand function, pain, or dissatisfaction with aesthetics and functionality. After six months, the score indicates improvements in the patient’s hand function, possibly in reducing pain, increasing functionality, or improving aesthetics and satisfaction. At the end of the year-long follow-up, a significant decrease in the overall score is highlighted, indicating that the patient has achieved a significant improvement in hand function.

Furthermore, we can calculate the percentage improvement in Brief Michigan Hand Outcomes Questionnaire (MHQ) scores between the various time points. Specifically: (i) from Month 1 to Month 6, the MHQ score improved by approximately 36.73%; (ii) from Month 6 to Month 12, the MHQ score improved by about 41.94%; (iii) from Month 1 to Month 12, the MHQ score improved overall by approximately 63.27%. Although statistical significance cannot be determined with just one patient, significant observations can still be made. The initial six months improvement is remarkable, suggesting that the surgery had a rapid and positive effect on the patient’s hand function. The continuing higher percentage of improvement from month 6 to month 12 indicates sustained and significant progress.

Overall, a 63.27% improvement in one year is highly significant. This indicates a substantial reduction in pain, enhanced hand function, or both. The sustained improvement over time suggests that the results are lasting.

Additionally, the patient was evaluated for other clinical outcomes such as strength using the Jamar test, pain using the Visual Analog Scale (VAS), and sensitivity using the Weber test (Table 2). Jamar Test [11,12] is a standardized test often used to measure grip strength, involving the use of a dynamometer to record the force exerted when squeezing the handle. Visual Analog Scale (VAS) is a widely used tool to measure pain intensity pain [13,14], involving a 10 cm line where one end signifies ‘no pain’ and the other end represents ‘worst pain imaginable’. Participants mark a point on the line that corresponds to their perceived level of pain, which can then be quantified by measuring the distance from the ‘no pain’ end. Weber Test [15,16] evaluates sensitivity, specifically the ability to discern two points pressed onto the skin. It helps assess the tactile discrimination threshold, which can indicate nerve function issues. We evaluated, also, the range of motion using Total Active Motion measurement (TAM). TAM considers the hand’s metacarpophalangeal (MCP), proximal interphalangeal (PIP), and distal interphalangeal (DIP) joints according to the formula: MCP + PIP + DIP of the affected digit minus the sum of the extensor lag of (MCP + PIP + DIP) of the same digit [17], classifying TAM as “excellent” if normal, “good” if TAM greater than 75% of the normal side, “poor” if TAM less than 50% of the normal side and “worse” if TAM was worse than before surgery. We named this value as the “percentage of range motion (%ROM)”. The %ROM data reflect hand function over time, resulting as “poor” at 1 month, “good” at 6 months, and “excellent” at 12 months. While statistical analyses are not feasible with one subject and qualitative data, a clear improvement in hand function is observed across the three time points. The progression from “poor” to “excellent” suggests significant recovery.

**Figure 5:**
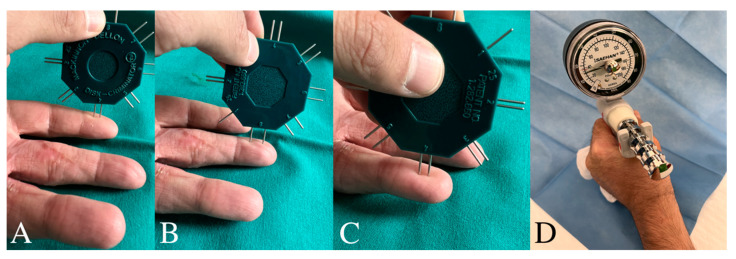
Weber and Jamar Test at 1 year of follow-up. (**A**). II finger 4 mm; (**B**). III finger 6 mm; (**C**). IV finger 4 mm; (**D**). Jamar 24 kg.

By examining the data, a clear trend towards a reduction in the two-point discrimination distance (Weber test) over time can be observed, indicating an improvement in sensitivity (Figure 5A–C). Analyzing the percentage of improvement between different measurements, statistical analysis shows that sensitivity consistently improves, with a 50% reduction in discrimination distance every six months, suggesting a progressive improvement in sensory function over the course of a year.

Analyzing the data regarding grip strength through the Jamar test (Figure 5D), the improvement trend shows that from the first to the sixth month, grip strength increased by 140%, while in the following six months, grip strength further increased by 100%. This suggests that after an initial rapid improvement, strength continues to improve significantly from the first to the second semester. The constant and significant increase in strength suggests that the rehabilitation program and healing process have progressed very well.

**Figure 6:**
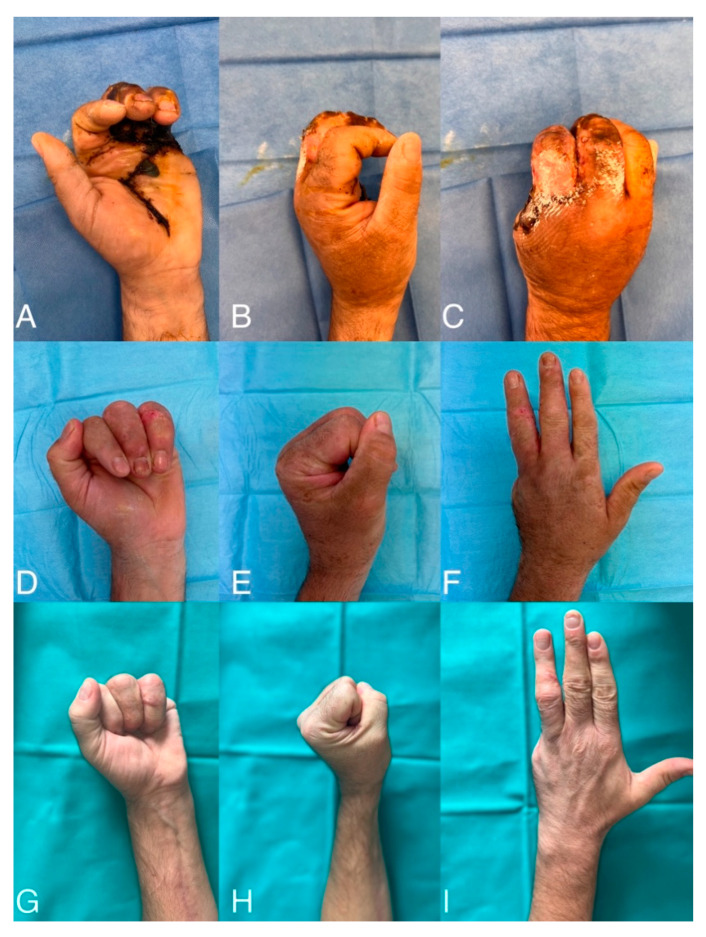
Range of Motion during follow-up period (**A**–**I**). Clinical presentation at one month (**A**–**C**). Clinical presentation at 6 months (**D**–**F**). Clinical presentation at one year (**G**–**I**).

Regarding pain measured by VAS, it is evident that pain decreased by 71.43% in the first six months after surgery, while no further improvement is evident between six and twelve months, as the score remains stable. This suggests that the level of pain achieved at six months was maintained up to twelve months post-intervention. These data indicate success in the control of post-operative pain, with significant relief within six months and lasting stability of improvement up to twelve months. Figure 6 shows the improvement in clinical functionality during the follow-up period.

The data show the range of motion (ROM) of the hand at 1 month (30°), 6 months (190°), and 12 months (250°) for a single subject. A clear improvement is observed over time. The increase in ROM is 160° between 1 and 6 months and 60° between 6 and 12 months.

## 3. Discussion

Digit amputations, but also digit hypoperfusion, are some of the most common traumas that are taken care of by hand surgeons in the emergency department. Although occupational injuries are decreasing due to the greater number of safety control devices, home accidents and trauma during recreational activities remain high in number, especially in agricultural settings.

The diagnostic–therapeutic process should be different when facing a complete amputation or an incomplete amputation where skin bridges are present, especially during the time between the trauma and the reconstructive surgery.

A clean detachment of the amputated stump is a clear trauma to be centralized in a Hand Surgery Hub Center; whereby delays in arrival at the referral center may be due to logistical problems related to patient, transport, or resources available in the referring hospital.

Instead, the incomplete amputations of hands or digits could be underestimated by untrained eyes as open fractures because residual circulation occurs through the remaining viable bridging structures. Wounds are either sutured urgently and monitored in the following days or scheduled for elective surgery to repair tendons and bone fractures. In this setting, as the hours go by, a gradual discoloration could appear and the patient is centralized in a Hand Surgery Center even days after injury, after a prolonged warm ischemia time. The clinical case reported in this article is a typical example of neglected vascular injuries in sub-amputation trauma. Woo and colleagues showed 12 cases of delayed revascularization after prolonged warm hypoperfusion time (mean warm ischemia time 53 h) due to neglected vascular injuries associated with incomplete amputations [18]. They concluded that there should be no hesitation in attempting revascularization in these patients, with a maximum delay of 72 h being acceptable.

An anatomical consideration is mandatory since digits have a small amount of tissue mass. Thus, the replanted or revascularized digits are less likely to develop systemic toxemia after prolonged warm ischemia or prolonged hypoperfusion condition. The greatest risk is the no-reflow phenomenon. Moreover, an increased risk of joint stiffness was described as well as an increment in cold intolerance compared with immediate replantation [18,19].

However, the role of ischemia time as predictive factor for microsurgery success is controversial and debated. Some authors affirmed that ischemia time is not related to digit survival and outcome [20,21,22], while others found a statistically significant relationship between ischemia time and success rate [23,24,25]. An interesting finding emerged from a retrospective study by Navarro and colleagues [26]; they observed that ischemia time was statistically significant in replantation but not in revascularization.

In summary, our clinical experience describes the recovery of a patient after a delayed digital revascularization surgery. The constant improvements observed in MHQ scores, grip strength, sensitivity, pain reduction, TAM and %ROM suggest that the intervention was highly effective and that rehabilitation led to positive and lasting outcomes, with the most significant improvements observed in the first 6 months. This confirms, as largely known, that this time frame represents the most critical time in hand rehabilitation, achieving good results at 6 months and continuing improvement until 12 months.

These observations indicate that, even in cases of prolonged hypoperfusion conditions, favorable clinical outcomes can be achieved with advanced surgical techniques and adequate post-operative management. This case highlights the importance of continuous assessment and monitoring of patients to optimize long-term functional results and improve quality of life.

The observations and percentage improvements provide a detailed overview of the recovery process, highlighting how a delayed intervention after 72 h from trauma and a well-structured rehabilitation program can lead to significant clinical benefits. While our case demonstrates a successful outcome, it is important to acknowledge the potential risk associated with delayed revascularization. Delays can lead to complications such as infection, increased joint stiffness, prolonged recovery times, and in some cases, failure of the digit revascularization due to the “no-reflow” phenomenon, where blood fails to re-enter the microcirculatory system. Studies [19,26] have highlighted cases where prolonged hypoperfusion times led to less favorable outcomes, underlining the importance of timely and appropriate intervention. Clinicians should weigh the benefits of delayed procedures against these risks, and continuous monitoring and adjustments in therapy should be made to mitigate potential adverse effects.

Finally, a technical and economic consideration should be obligatory. Digital replantation or revascularization is a highly demanding surgery in terms of surgical skills and health resources. Precision, accuracy and speed of execution make the difference in the results. Moreover, the microsurgery technique requires qualified, free-from-fatigue and trained surgeons. All these requirements cannot be present during night shifts. Furthermore, an overnight operation costs the health care system much more than a daily surgery.

Given all these considerations, several authors proposed different management of amputations; they advanced the overnight-delayed replantation [26,27]. This strategy resulted in lower operating room costs and rested and better-performing staff.

## 4. Conclusions

Despite a large discrepancy within the literature about functional scoring, outcome evaluation, and calculation of ischemia time and regardless of the different inherent peculiarities of each national health system, more and more evidence showed that long ischemia time, as well as prolonged hypoperfusion time, are well tolerated and delayed revascularizations have functional results superimposable to immediate surgery. Although it is not possible to draw definitive conclusions from a single case report, it inevitably prompts reflection on the management of these emergencies. Thus, we propose an “elective-urgence” schedule in a dedicated operating room active daily throughout the week. This approach should ensure the best surgical condition to maximize the results and minimize costs.

## Figures and Tables

**Table 1:** Brief Michigan Hand Outcomes Questionnaire (MHQ).

MHQ	T1	T6	T12
How well did your hand(s) work during the past week?			
(*Function: 1 very good to 5 very poor*)	5	3	1
How was the sensation (feeling) in your hand(s) during the past week?			
(*Function: 1 very good to 5 very poor*)	5	3	1
How difficult was it for you to hold a frying pain during the last week?			
(*Daily activities: 1 not at all difficult to 5 very difficult*)	5	3	1
How difficult was it for you to button a shirt or blouse during the past week?			
(*Daily activities: 1 not at all difficult to 5 very difficult*)	5	3	1
In the past 4 weeks, how often were you unable to do your work because of problems with your hand(s)/wrist(s)			
(*Work activities: 1 always to 5 never*)	3	2	1
In the past 4 weeks, how often did you take longer to do tasks in your work because of problems with your hand(s)/wrist(s)			
(*Work activities: 1 always to 5 never*)	4	3	1
How often did the pain in your hand(s)/wrist(s) interfere with your daily activities?			
(*Pain: 1 very mild to 5 very severe*)	4	1	1
Describe the pain in your hand(s)/wrist(s) in the past week			
(*Pain: 1 very mild to 5 very severe*)	4	1	1
I am satisfied with the look of my hands			
(*Aesthetics: 1 strongly agree to 5 strongly disagree*)	3	3	3
The appearance of my hands interferes with my normal daily activities			
(*Aesthetics: 1 strongly agree to 5 strongly disagree*)	5	5	5
In the past week, how satisfied were you with the motion of your fingers?			
(*Satisfaction: 1 very satisfied to 5 very dissatisfied*)	4	3	1
In the past week, how satisfied were you with the motion of your wrist?			
(*Satisfaction: 1 very satisfied to 5 very dissatisfied*)	2	1	1
**Total**	**49**	**31**	**18**

**Table 2:** Clinical Outcomes Before and After Surgery.

Clinical Examination Test	T1	T6	T12
Weber Test	>15 mm	8 mm	4 mm
Jamar Score	5 kg	12 kg	24 kg
Visual Analog Scale (VAS) score	7	2	2
TAM	30°	190°	250°
%ROM	Poor	good	excellent

## Data Availability

Data are contained within the article.
